# Factors associated with hindering the acceptance of HPV vaccination among caregivers - A cross-sectional study in Argentina

**DOI:** 10.1371/journal.pone.0229793

**Published:** 2020-03-10

**Authors:** Raúl Martín Chaparro, Belén Rodríguez, Yanina Maza, Daniela Moyano, Akram Hernández-Vásquez

**Affiliations:** 1 Universidad de Buenos Aires, Buenos Aires, Argentina; 2 Universidad Nacional del Nordeste, Chaco, Argentina; 3 Escuela de Nutrición, Facultad de Ciencias Médicas, Universidad Nacional de Córdoba, Córdoba, Argentina; 4 Centro de Excelencia en Investigaciones Económicas y Sociales, Vicerrectorado de Investigación, Universidad San Ignacio de Loyola, Lima, Peru; University of Campania, ITALY

## Abstract

**Background:**

The northeastern region of Argentina has the highest age-adjusted cervical cancer mortality rates. Given the strong link between HPV infections and cervical cancer, one of the main interventions is the population-based use of HPV vaccines. However, the acceptability is not very clear in low- and middle-income countries. The purpose of this study was to estimate the level of HPV vaccine acceptance and associated determinants among caregivers of girls in a northeastern city of Argentina.

**Methods:**

A school-based survey was conducted in 2015 using a multistage sampling method. The primary sample unit were schools stratified by socioeconomic status selected at random, and caregivers of school girls were interviewed. The acceptability was determined using the adapted Theory of Planned Behavior. We performed logistic regression models to assess associated determinants.

**Results:**

The study included 347 caregivers. The intention to vaccinate was 59.88%. A positive attitude of caregivers (aOR 4.67; 3.11–7.03) and positive influence of social norms (aOR 1.95; 1.03–3.70) were the main predictors independently associated to the intention to vaccinate against HPV. In contrast, practicing a Christian non-Catholic religion decreased the intention to vaccinate against HPV (OR 0.59; 0.36–0.95). All other factors evaluated were not significantly associated with intention to vaccinate against HPV.

**Conclusions:**

This study shows that evaluating attitudes, normative social beliefs, and perceived self-efficacy regarding HPV vaccination can be of utmost importance for mapping and planning of health-related strategies in developing countries.

## Introduction

Cervical cancer (CC) is the fourth most common cancer in women worldwide [1]. The burden of the disease is higher in low- and middle-income countries, where CC accounts for approximately 12% of incident cancer cases in women, and 7.5% of cancer deaths. According to GLOBOCAN, around 4,500 new CC cases and 2,200 CC deaths occur in Argentina every year [2]. There is an important inequality in the distribution of CC burden of disease in Argentina, where areas of lower socioeconomic level have the highest mortality rates of the country. For example, the province of Chaco in the northeastern region ranks third among those with the highest age-standardized mortality rates in the country -just under 12 deaths per 100,000 women- as reported by the Argentinian National Cancer Institute in 2016 [3].

Given the strong link between HPV infections and cervical cancer, several new interventions have been introduced to reduce the burden of disease. The main one is the population-based use of HPV vaccines. In Argentina, two HPV vaccines, *Cervarix* (which covers HPV types 16 and 18) and *Gardasil* (which covers HPV types 6, 11, 16, and 18), were approved in 2006 and 2007, respectively by the National Ministry of Health in Argentina [4, 5]. The bivalent vaccine was added to the national immunization schedule in 2011, only for 11-year-old girls born in 2000 or after [6]. Since 2014, the national immunization schedule includes the administration of two doses of free quadrivalent vaccine to 11-year-old girls, and as of 2017, it also includes 11-year-old boys born after 2006 [7]. It is worth noting that this study was conducted in 2016, when boys were not yet included in the immunization schedule.

Although the World Health Organization has urged countries to achieve at least an 80% of vaccine coverage in every district for all vaccines included in the national program of immunization [8], only 46% of eligible girls received the second dose of HPV vaccine in Chaco in 2015, well below the recommendations of WHO [9].

People´s intention to vaccinate against HPV is a factor that contributes to the acceptance of the vaccine. Thus, many studies have been carried out to research the determinants of HPV vaccine acceptance [10–13]. In this sense, the fact that HPV vaccines are aimed at adolescents raises the following issues: a) there may be important barriers to access preventive health services for this population subgroup, and b) caregivers opinions affect adolescents’ vaccination. The studies on this subject have been carried out in high-income countries, where potentially associated factors such as perception of risk, awareness and knowledge of HPV, and recommendation source, among others, have been assessed [13]. Latin America countries such as Colombia, Mexico, and Brazil have studied HPV vaccine acceptability, finding that it varied depending on the social context characteristics and that knowledge of the HPV vaccine was an important determinant for its acceptability [14–16]. However, a Peruvian study found high acceptability rates despite little knowledge of HPV or cervical cancer [17]. In Argentina, mothers’ acceptability of HPV vaccination was studied only in the city of Buenos Aires and before being incorporated to the National immunization schedule [18].

There is a lack of knowledge regarding how caregivers´ beliefs and sociodemographic factors could affect the individual behavior of vaccinating girls against HPV in Argentina. In accordance with the Theory of Planned Behavior [19, 20], which has been widely used to explore HPV vaccination behavior [21–24], the higher the intention the more likely to perform such behavior. Understanding the influences of caregivers´ intention to vaccinate could inform policy decision-making to bring about behavioral changes, especially in populations with the highest burden of disease. The aim of this study was to estimate the intention to vaccinate against HPV among caregivers of girls eligible to receive the vaccine in the city of Resistencia, Chaco, as well as to know its main predictors.

## Materials and methods

### Study setting, sampling, and data collection

The area of study was the city of Resistencia, capital city of the Chaco province, in Northwestern Argentina. Resistencia is the most populated city in the province and is the main economic and cultural center; it is also one of the most productive cities in the Northwestern region of Argentina [25]. It is located in the department of San Fernando and has 390,874 inhabitants [26]. Resistencia represents one the poorest locations in Northeastern Argentina with the highest mortality rate due to Cervical Cancer [3].

Parents or caregivers of 11-year-old girls attending 5th or 6th school grade were eligible to participate and recruited from June to December 2015 in the city of Resistencia, Chaco. A multistage sampling method was used considering an expected prevalence of intention to vaccine of 46.6% (based on a prior pilot study) for a universe of 3,190 11-year-old girls [27]. Considering an alpha error of 0.05 and a design effect of 1.5, the estimated sample size was 420 girls.

Schools within geographical segments of 300-houses (radius) from the 2010 Argentinean National Census were classified into five strata according to the proportion of unsatisfied basic needs (UBN) of each radius [28]. The UBN is an indicator usually used to measure and characterize structural poverty, and it is retrieved from the population and housing census [29]. Each stratum was assigned to a particular weight according to the number of schools it contained. Subsequently, given that an average of 38 girls attended each school between morning and afternoon, we randomly selected 21 schools (out of a total of 84 schools, both public and private) to reach the required sample size.

The selected schools were contacted by an investigator and invited to participate in the study. An informed consent form was sent to the parents or caregivers of eligible girls. Those who accepted it were sent a self-administered questionnaire by the school teachers. All teachers involved in the study were trained to supervise the completeness of the questionnaire by experienced researchers.

### Theoretical model

The survey was based on the theoretical model of Adapted Theory of Planned Behavior (TPB) [19, 20]. The central factor in the TPB is the intention to perform a given behavior and as a general rule, the stronger the intention to engage in a behavior, the more likely should be its performance. Furthermore, this model proposes that the intention to perform an act has three determinants: the attitude towards the act, the subjective norm, and the perceived behavioral control.

This well-established model used in other preventive strategies provided the basis for questionnaire development regarding healthy behaviors. For this survey, we examined caregivers´ intention to have 11-year-old girls vaccinated against HPV and various factors that predicted vaccine uptake.

### Questionnaire

Questionnaire development adhered to the steps needed to construct a TPB questionnaire and was based on a pilot study carried out to explore the intention to vaccinate, and to test and validate the questionnaire [27]. The target population was defined as parents or caregivers of 11-year-old girls attending 5th or 6th school grade in Resistencia city in 2015.

### Outcome variable

The outcome being evaluated was caregivers´ intention to vaccinate their 11-year-old daughters against HPV at the moment of filing the questionnaire. Intention was measured by parents or caregivers self-report on their daughters´ HPV vaccination under the statement: “*I have the intention to have my daughter vaccinated”*. Parental intention was measured using a 5-point Likert scale and dichotomized using the mean score as a cut-off value. This method has been reported in other studies [21,22].

### Predictors of intention to HPV vaccination

Caregivers were asked to agree or disagree with several statements about HPV virus, related diseases and vaccination, using a 5-point Likert scale where 1 meant to strongly disagree; 3 meant neutral; and 5 meant to strongly agree. Afterwards, these statements were classified into TPB´s Attitude, Subjective Norm and Perceived control domains, which were the main predictors under assessment.

Attitudes domain refers to the positive or negative judge a person makes when performing or not a behavior. In this study, it explored HPV virus awareness and knowledge as a necessary factor for cervical cancer development, as well as of the HPV vaccine effectiveness for cervical cancer prevention. The attitude domain scale of 3 items used a cut-off point of > = 4.31 (mean) to classify attitudes towards HPV vaccination as “positive”. Subjective norms domain refers to the beliefs about the perspective of significant people regarding weather or not a behavior is recommended. In this case, it explored recommendations to vaccinate against HPV by family members, close friends, teachers, physicians, nurses or television, and religion practices. Subjective norm scale consisted of 4 items, and a cut-off point of > = 3.73 (mean) was used to classify it as positive (meaning that Subjective norm supported HPV vaccination). Finally, Perceived control domain, also known as “self-efficacy” to vaccinate consisted of a 6 items scale, and a cut-off point of > = 3.65 (mean) was used to consider it positive.

Because the wording of statements was sometimes positive and sometimes negative, we reversed the scoring where needed so that a higher score indicated always a strong agreement with HPV vaccination. Using the mean score as a cut-off value is an accepted method and has been used in other studies [21, 22].

### Covariates

As explanatory variables, we considered socio-demographic characteristics including age (in years), gender (male and female), family role (mother, father and others -which included grandparents or legal guardians), educational attainment (none or primary, secondary, tertiary or university), main occupation (full-time employment, self-employed, informal worker and retired), religion practice (yes/no), and type of religion (Catholic and other Christian-Non Catholic religions). Other covariates assessed were “knowledge about the HPV virus” (yes/ no), “family history of cervical cancer” (yes/ no), “advice to vaccinate by a teacher (yes/ no), pediatrician or nurse” (yes/ no), and “overall adherence to the national vaccination schedule” (yes/ no) [30].

### Data analysis

We summarized continuous variables using means with standard deviations and categorical variables with percentages and 95% Wald Confidence Intervals (CI). To test the statistical significance of the difference between the intention to vaccinate of each categorical variable, the χ2 test was applied. T-test was used when testing continuous variables. Backward stepwise logistic regression analysis was used to study the effect of independent variables on the intention to the HPV vaccine. A p-value <0.2 was required for retention of each variable in the final model. Multicollinearity of independent variables was checked by the variance inflation factor (VIF) statistic. Results are presented as crude (ORc) or adjusted odds ratio (aOR) and 95% confidence intervals (CI). The Area Under the Curve (AUC) and Hosmer-Lemeshow goodness of fit tests were considered as discrimination and calibration parameters for the model. All statistical analyses were performed using the statistical software Stata IC, version 14.2 (Stata Corp., College Station, TX, USA). Due to the complex sampling design, we used the *svyset* command to account for sample stratification and weighting to provide unbiased estimates of the population parameters. Statistical significance was defined as a two-sided p-value <0.05 for all tests.

### Ethics statements

The protocol was independently reviewed and approved by the Bioethics Committee of the Faculty of Medicine of the *Universidad Nacional del Nordeste*, Argentina (Resolution 30/14, adopted in December 2014). Written informed consent was provided by all participants.

## Results

**Fig 1:**
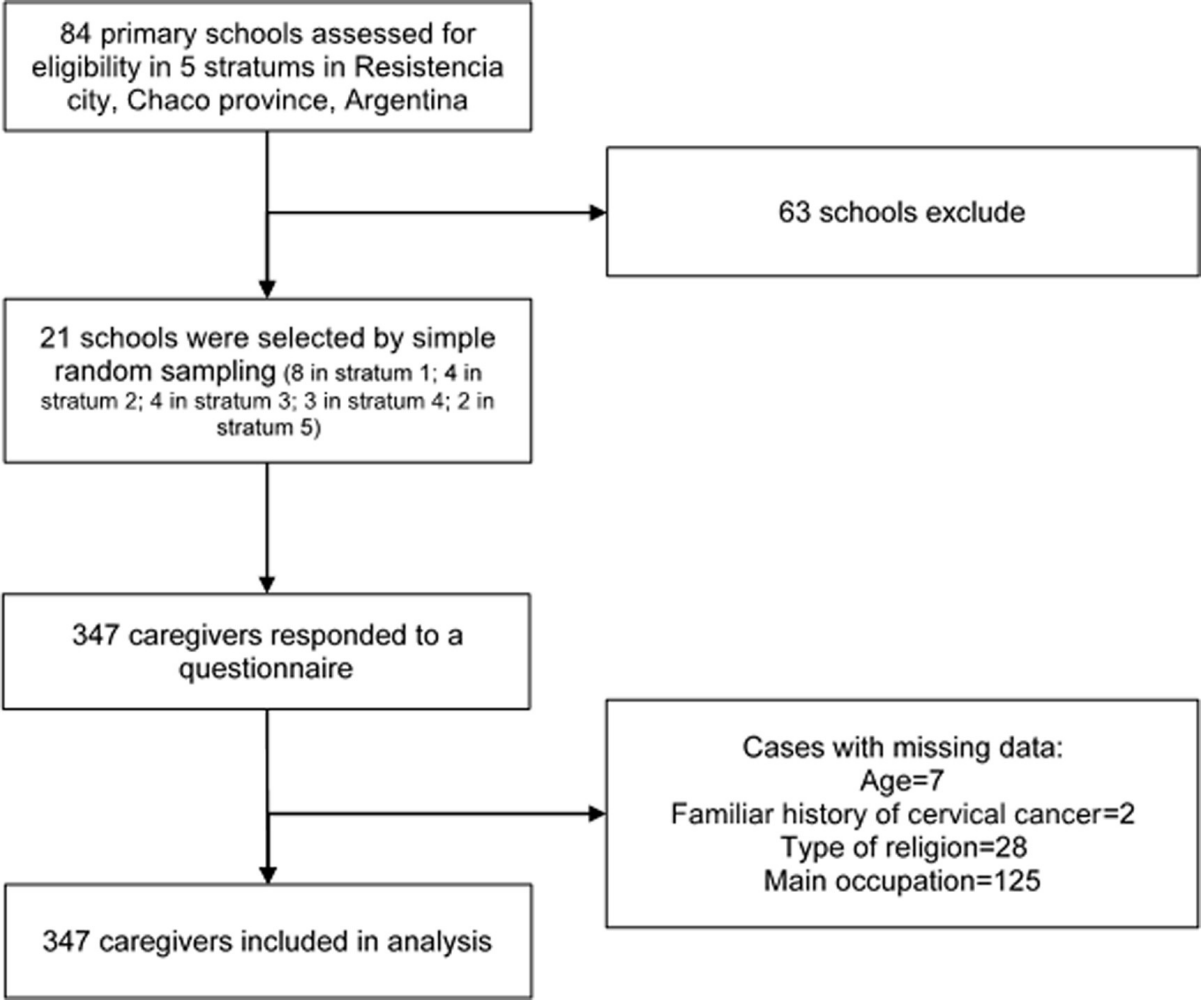
Flow chart for the selection of study participants.

The flow chart for the participants’ inclusion is shown in ***Fig 1.***

The socio-demographic characteristics of the 347 parents/caregivers are summarized in ***Table 1***. The response rate was 79%.

**Table 1:** Baseline socio-demographic characteristics of caregivers (n = 347).

Variable	All participants (n = 347)	Weighted proportion*	
	N	%	(95% CI)
Mean age, years**	39,0±0,51		
Family role			
Mother	290	83.7	(80.9–86.1)
Father	44	12.5	(10.2–15.2)
Other	13	3.8	(2.1–7.0)
Gender			
Female	303	87.5	(84.8–89.8)
Male	44	12.5	(10.2–15.2)
Educational attainment			
None/Primary	86	24.4	(17.6–32.8)
Secondary	150	42.6	(37.0–48.4)
Tertiary/University	111	33.0	(23.8–43.7)
Main occupation			
Full-time employment	97	43.7	(36.6–51.0)
Informal worker	39	17.6	(13.2–23.1)
Self-employed	42	19.6	(13.8–27.1)
Not employed	36	15.7	(10.0–23.8)
Retired	8	3.4	(2.0–5.8)
Practice some type of religion			
No	32	9.4	(6.5–13.3)
Yes	315	90.6	(86.7–93.5)
Type of religion			
Catholic	220	70.8	(65.3–75.7)
Other Christian religions	95	29.2	(24.3–34.7)
Knowledge about the HPV virus			
No	40	11.1	(7.5–16.2)
Yes	307	88.9	(83.8–92.5)
Family history of cervical cancer			
No	291	84.0	(78.7–88.3)
Yes	54	16.0	(11.7–21.3)
Recommendation to vaccinate by pediatrician			
No	99	27.6	(24.2–31.2)
Yes	248	72.4	(68.8–75.8)
Recommendation to vaccinate by nurse			
No	133	38.5	(32.7–44.7)
Yes	214	61.5	(55.3–67.3)
Recommendation to vaccinate by teacher			
No	225	65.1	(57.1–72.3)
Yes	122	34.9	(27.7–42.9)

Results may not add due to missing values.

*Proportions are weighted according to sampling specifications.

**Data expressed as mean ± SD.

CI confidence interval.

The reported intention to vaccinate was 59.88% (95% CI: 57.57–62.14). The mean age of the caregivers surveyed was 39 years (SD 0.51). According to baseline socio-demographic characteristics, most of them were mothers (83.7%), had a tertiary/university (33.9%) level of education, and full-time employment (43.7%). The majority of the participants were Catholics. Recommendation to vaccinate by pediatrician and nurse were similar but higher than the recommendation by teacher.

Mean scores within TPB domains and items used as predictors are shown in ***Table 2***. All domains presented an acceptable Cronbach Alpha. The items with higher variability were found in subjective norm and perceived behavioral control domains.

**Table 2:** Mean (SD) of items evaluated within TPB domains, and Cronbach alpha of each domain.

Attitudes toward HPV vaccine	Mean (SD)
*Cervical cancer is a serious disease*	4.53 (0.64)
*Vaccines prevent cervical cancer*	4.20 (0.77)
*HPV vaccine is effective against cervical cancer*	4.23 (0.85)
Cronbach alpha	0.64
**Subjective norms**	
*My religious practices allow me the vaccination*	3.80 (1.20)
*My family recommends the vaccination*	3.52 (1.32)
*Influential people get their daughters vaccinated*	4.19 (0.88)
*People closed to me and whose opinion I value approve the vaccination against HPV*	3.40 (1.24)
Cronbach alpha	0.66
**Perceived behavioral control**	
*It is possible that my daughter receives HPV vaccine*	4.45 (0.72)
*It is a parental decision to vaccinate their daughter*	3.44 (1.29)
*I will do my best to vaccinate my daughter against HPV*	3.92 (1.12)
*I am sure my daughter would receive the HPV vaccine*	3.48 (1.29)
*Other duties do not interfere with my daughter receiving the HPV vaccine*	3.41 (1.38)
*I would have my daughter vaccinated even if it was not mandatory to attend school*	3.21 (1.42)
Cronbach alpha	0.74

***Table 3***presents the results of the regression models with their crude (ORc) and adjusted (aOR) odds ratios. Attitudes towards the HPV vaccine in particular was the most important TPB domain related to the intention to vaccinate reported by the caregivers surveyed (ORc 4.58; CI 95%: 3.14–6.69). It was followed by subjective norm (ORc 2.07; CI 95%: 1.22–3.53) and perceived behavioral control (ORc 1.95; CI 95%: 1.20–3.15). A positive attitude of caregivers (aOR 4.67; *3*.*11–7*.*03*) and positive influence of social norms (aOR 1.95; *1*.*03–3*.*70*) were the main predictors independently associated to the intention to vaccinate against HPV. In contrast, practicing a Christian non-Catholic religion decreased the intention to vaccinate against HPV (OR 0.59; *0*.*36–0*.*95*). All of the other factors evaluated were not significantly associated with intention to vaccinate against HPV.

**Table 3:** Factors associated with intention to HPV vaccination by caregivers (N = 347).

Variable	Model 1^a^			Model 2^b^			Model 3^c^		
	cOR	(95% CI)	P	aOR	(95% CI)	p	aOR	(95% CI)	p
**Positive attitude**	4.58	(3.11–6.75)	<0.001	4.50	(2.91–6.96)	<0.001	4.67	(3.11–7.03)	<0.001
**Non-positive attitude**	1	-	-	1	-	-	1	-	-
**Positive influence**	1.99	(1.04–3.79)	0.037	2.06	(1.06–4.03)	0.034	1.95	(1.03–3.70)	0.041
**Non-positive influence**	1	-	-	1	-	-	1	-	-
**Self-controlled**	0.71	(0.42–1.20)	0.201	0.70	(0.41–1.21)	0.201	0.69	(0.41–1.15)	0.156
**Not self-controlled**	1	-	-	1	-	-	1	-	-
**Gender**									
Female	1	-	-	1	-	-	1	-	-
Male	1.30	(0.76–2.20)	0.335	0.67	(0.36–1.24)	0.204	0.69	(0.37–1.30)	0.250
**Age, years**	1.03	(1.00–1.06)	0.067	1.00	(0.97–1.04)	0.981	1.00	(0.97–1.03)	0.817
**Caregivers’ educational attainment**									
None/Primary	1	-	-	1	-	-	-	-	-
Secondary	2.98	(1.62–5.48)	<0.001	1.15	(0.58–2.27)	0.685	-	-	-
Tertiary/University	4.02	(2.10–7.70)	<0.001	1.00	(0.40–2.49)	0.992	-	-	-
**Type of religion**									
Catholic	1	-	-	1	-	-	1	-	-
Christian non-Catholic	0.50	(0.33–0.77)	0.001	0.58	(0.35–0.95)	0.032	0.59	(0.36–0.95)	0.031
**Knowledge about the HPV virus**									
No	1	-	-	1	-	-	-	-	-
Yes	2.60	(1.09–6.18)	0.031	0.49	(0.12–1,96)	0.313	-	-	-
**Family history of cervical cancer**									
No	1	-	-	1	-	-	-	-	-
Yes	1.22	(0.60–2.51)	0.584	1.09	(0.43–2.76)	0.859	-	-	-
**Advice to vaccinate by pediatricians**									
No	1	-	-	1	-	-	-	-	-
Yes	1.74	(1.10–2.74)	0.017	1.03	(0.60–1.76)	0.925	-	-	-
**Advice to vaccinate by nurses**									
No	1	-	-	1	-	-	-	-	-
Yes	1.19	(0.75–1.87)	0.462	1.11	(0.63–1.97)	0.710	-	-	-
**Advice to vaccinate by teachers**									
No	1	-	-	1	-	-	-	-	-
Yes	1.52	(0.99–2.35)	0.057	1.23	(0.71–2.14)	0.459	-	-	-

OR: Odds Ratio.

CI: confidence interval.

a Crude model;

b Full model with all variables;

c Final model with significant variables.

## Discussion

This study sought to explore factors influencing caregivers’ intention to vaccinate their 11-year-old daughters in Resistencia, province of Chaco (Argentina) using the Theory of Planned Behavior. Our results showed an intention to vaccinate against HPV of 59%. Similar findings were also reported from Italy, with the intention to vaccinate with HPV was 61.8% [31]. A positive attitude towards HPV vaccine and positive subjective norm were the most influential predictors associated with caregivers’ intention to vaccinate against HPV. Furthermore, these potentially modifiable factors could be useful health-policy drivers in order to shape and modify individual behavior and public health outcomes.

In accordance with the results of similar studies, most of the caregivers surveyed were women of around 40 years of age (90%) [16, 32]. This is to be expected because women have been traditionally in charge of caring for and making decisions regarding their children’s health [33–35].

A qualitative systematic review published in 2015 assessed the use of various theories applied to the understanding of HPV vaccination as a behavior [36]. Fifteen out of 31 studies used TPB as framework, making TPB the most frequently used theory. In accordance with our findings, attitudes and subjective norms were positively associated with the intention to vaccinate against HPV across all 15 studies. Perceived behavior control (PBC) was significantly associated only in half of the studies included, becoming the most controversial domain in TPB.

More recent studies also support both attitudes and subject norms domains as the main predictors of HPV vaccination intention. Wang *et al*. studied parental intention to vaccinate young girls in Taiwan, and found significant association for all three domains, including PBC. However, the study was based on a convenience sample which could limit its generalizability to other contexts [33]. Kim *et al*. evaluated mothers’ intention to vaccinate their children in South Korea, and found controversial results regarding PBC associations with the intention to vaccinate against HPV (PBC was significantly associated in boys but not in girls) [21]. Further research may be needed to standardize the items studied under the construct of PBC and to assess its impact on the intention to vaccinate and ultimately performing the actual behavior.

Other researchers have studied different social and demographic factors, such as educational attainment, socioeconomic status and religion practices of caregivers. Our findings only support a negative association between practicing a Christian non-Catholic religion and the intention to vaccinate. Galbraith *et al*. found that non-Baptists were 3.6 times more likely to accept the HPV vaccine for their daughters compared to Baptists [37]. Shelton *et al*. found that Catholic parents were more likely than non-religious parents to have their daughters vaccinated [38]. Similarly to the results of Shelton *et al*., our study showed that Catholic caregivers were more likely to accept the HPV vaccine. The low rate of acceptance among non-Catholic parents could be due to sexually-related concerns (22).

Contrary to our findings, other studies in high income countries have found that HPV knowledge and fear of adverse events could be important barriers to HPV vaccine uptake [32, 39–41]. Ogilvie *et al*. found that in British Columbia, Canada, the main reason for having girls vaccinated was the vaccine’s effectiveness, while the main reasons for not having girls receive the vaccine were concerns about its safety, preference to wait until girls were older, and not enough information to make informed decisions [32]. VanWormer *et al*. assessed how changes in parents’ attitudes were associated with improvements in HPV vaccine uptake in the United States [40]. Despite acceptability being relatively high, parental concerns about the HPV vaccine’s safety appeared to be the most important barrier.

Despite the positive association between subjective norms and intention to vaccinate, our findings showed that neither the recommendation to vaccinate made by a physician, nor a nurse or teacher were relevant to caregivers’ intention to have their daughters vaccinated against HPV. This is supported by other studies which have found similar results. For example, VanWormer *et al*. showed that neither physicians’ nor nurses’ recommendations to get the HPV vaccine in the previous year were associated with the actual receipt of the next HPV vaccine dose [40]. It is worth noting that in our study, only 72% of caregivers reported to have been advised by physicians to vaccinate their daughters. However, these results may be limited by recall bias and by the fact that our study did not enquire about the frequency or strength of the physician advice. Nonetheless, other studies that have researched directly on physicians´ attitudes and practices towards HPV vaccines have found similar or even lower results [42–44], indicating that this could be an important area to further explore in the future towards improving HPV vaccine coverage by reducing the number of “missed opportunities” to address HPV vaccination with caregivers.

We believe that these results could be relevant when putting the issues that are the object of our research into the Latin American context, particularly in South America. It is very important to understand the factors that may interfere with or enhance the success of HPV immunization programs worldwide, and especially in developing countries such as Argentina. Our findings suggest that government efforts towards improving HPV vaccination uptake among girls in Resistencia, Chaco should focus primarily in the implementation of strategies to shape a positive attitude towards HPV vaccination. Subjective norms also increased the intention to vaccinate, but only when family members, influential or close people were considered.

Our study may have limited generalizability beyond the caregivers living in Resistencia, Chaco given that the individual constructs under evaluation could potentially vary by different cultures and social context. However, efforts were made to improve generalizability by means of random sampling schools. A cognitive bias could happen to understand negative statements under TPB although the instrument was pretested with a small sample of similar population. Another important limitation is the fact that our study did not include a qualitative assessment of the reasons why caregivers and adolescents didn’t have the intention to vaccinate, remaining a topic for further investigation in the Argentinean context.

## Conclusions

Evaluating attitudes, normative social beliefs and perceived self-efficacy regarding HPV vaccination can be of utmost importance for mapping and planning of health-related strategies in developing countries.

## Supporting information

S1 Table Database. (XLS)

S2 Table Codebook. (XLS)
